# Bond Relationship of Carbon Fiber-Reinforced Polymer (CFRP) Strengthened Steel Plates Exposed to Service Temperature

**DOI:** 10.3390/ma14133761

**Published:** 2021-07-05

**Authors:** Hui Li Lye, Bashar S. Mohammed, Mohamed Mubarak Abdul Wahab, Mohd Shahir Liew

**Affiliations:** Department of Civil and Environmental Engineering, Universiti Teknologi PETRONAS (UTP), Bandar Seri Iskandar 32610, Perak, Malaysia; hui_17010162@utp.edu.my (H.L.L.); mubarakwahab@utp.edu.my (M.M.A.W.); shahir_liew@utp.edu.my (M.S.L.)

**Keywords:** carbon fiber-reinforced polymer (CFRP), adhesive, service temperature, tensile and flexural bonding, response surface methodology (RSM), strengthening

## Abstract

Emerging as a new technology, carbon fiber-reinforced polymer (CFRP) has been introduced to rehabilitate and strengthen steel structures using an adhesive agent. However, the outdoor service temperature is potentially degrading to the mechanical strength of the adhesive, as well as affecting the bonding of the strengthened steel structure. Therefore, this paper aims to investigate the bond relationship of CFRP-strengthened steel plates exposed to service temperatures. Two types of experiments were conducted to determine the tensile and flexural performance of CFRP-strengthened steel plates. The experiments were designed using a Box–Behnken design (BBD) and response surface methodology (RSM) by considering three parameters: service temperature (25 °C, 45 °C and 70 °C), number of CFRP layers (one, three and five layers) and bond length (40, 80 and 120 mm). The findings show the dominant failure mode transformed from adhesion failure between steel and adhesive interfaces to adhesion failure between CFRP and adhesive interfaces as the service temperature increased. The tensile strength improved by 25.62% when the service temperature increased. Field emission scanning electron microscope (FESEM) analysis proved that the strength enhancement is due to the densification and reduction of the adhesive particle microstructure gaps through the softening effect at service temperature. However, service temperature is found to have less impact on flexural strength. Incorporating the experimental results in RSM, two quadratic equations were developed to estimate the tensile and flexural strength of CFRP-strengthened steel plates. The high coefficient of determination, R2, yields at 0.9936 and 0.9846 indicate the high reliability of the models. Hence, it can be used as an estimation tool in the design stage.

## 1. Introduction

The deterioration of steel structures due to environmental exposure means that they are in need of being rehabilitated and strengthened to maintain and enhance structural integrity. The conventional retrofitting methods are the external attachment of a new steel plate or replacement with a new steel plate through welding or bolting. However, these methods require heavy lifting equipment, more labor, time for installation and higher fatigue stress and danger, particularly in high-elevation and fire-sensitive environments. Therefore, an alternative method is introduced in this paper: using carbon fiber-reinforced polymer (CFRP) with an adhesive agent. The outstanding characteristics of CFRP include low weight, high strength, stiffness, flexibility, resistance to corrosion and ease of installation, all of which are reasons for engineers to adopt it in civil industry [1,2,3]. Epoxy resin is an adhesive agent that is commonly used for the bonding of CFRP to structures. The high bond strength and excellent bond efficacy helps in stress transfer between the interfaces, and the best option is to attach the CFRP to the structure. However, the drawback of epoxy resin is its sensitivity to elevated temperature. Generally, epoxy resin possesses a low glass transition temperature ($T_{g}$). When temperature exceeds the $T_{g}$ at 50 °C, the adhesive mechanical properties transform from an elastic glassy state to a rubbery state, which significantly reduces the elastic modulus, stiffness and strength of the adhesive [4,5]. This situation strongly affects the bonding interfaces and adversely affects the bonding performance of CFRP-strengthened steel structures [6].

Much research has been conducted to understand the behavior of CFRP-strengthened steel plates exposed to elevated temperatures [7]. Nguyen, et al. [8] thermally soaked CFRP-strengthened steel plates in an environment chamber for 90 min at elevated temperature after curing for 10 days at room temperature. The outcome showed that the reduction in ultimate strength and stiffness with temperature is correlated to the shear strength of the adhesive. In addition, Lu, et al. [9] strengthened CFRP for single-sided and double-sided bonding on steel and exposed it to elevated temperatures, ranging from 25 to 120 °C for 20 min. The study showed that only 44.3% of the ultimate load capacity was left when the temperature reached 60 °C. Li, et al. [10] observed that, when the temperature exceeded $T_{g}$, the reduction in volume changed in its failure mode from debonding between the steel and adhesive interface to debonding between the CFRP and the adhesive interface. This significantly reduced the initial stiffness, load-carrying capacity and interfacial energy when the temperature approached $T_{g}$.

Furthermore, He, et al. [11] exposed the specimens for 160 min at temperatures ranging from 23 °C to 75 °C. The degradation of the adhesive elastic modulus led to a reduction in bond strength by 10% at $T_{g}$− 15 °C and 70% when $T_{g}+$15 °C. However, Chandrathilaka, et al. [12] tested eighty-two CFRP/steel double strap joints and reported that the reduction of bond strength, Poisson’s ratio and elastic modulus of CFRP/steel joint occurred in a similar manner. Chandrathilaka, et al. [13] also found that, despite the type and thickness of CFRP, an elevated temperature caused the initial softening of the bond and substantially lowered the shear stress to 10 MPa when the temperature exceeded 90 °C. Similar behavior was observed by Zhou, et al. [14], who found that the elastic modulus and initial stiffness of the bond-slip curve and the peak bond shear stress deteriorated with increasing temperature. However, the total fracture energy increased when temperature is below $T_{g}$ and then decreased when the temperature exceeded $T_{g}$. Nevertheless, Zhou, et al. [15] argued that the geometrical properties of bonded joints’ particularly insufficient bond length considerably affected the bond strength of the CFRP-strengthened steel plates.

However, some conflicting reports have been observed in adhesive properties when it is exposed to continuous elevated temperature, which is also known as service temperature. Qin, et al. [16] found that the tensile strength of adhesive increased by 27.55% when exposed under 80 °C for 10 days. This implies that the ductility and toughness of adhesive increases with longer temperature exposure [17]. Hu, et al. [18] found that, at service temperature, the internal stress of the adhesive began to release, and the adhesive molecules became more flexible and turned into a higher-degree cross-linking adhesive. To some extent, it could offset the strength degradation and increase the bond strength between the interfaces. Michels, et al. [19] also claimed that adhesive strength developed with age. Therefore, it is too conservative to test the specimen at a young age, as compared to later usage in practice, from both an engineering and economic point of view. The age at testing should be at least 14 days, instead of immediate testing after being exposed for only about an hour of elevated temperature.

This paper aims to investigate the bond relationship of CFRP-strengthened steel plates exposed to service temperatures. Response surface methodology (RSM) was used to generate the experiments by taking into account the parameters, including bond length (40, 80 and 120 mm), CFRP layers (one, three and five layers) and temperature (25 °C, 45 °C and 70 °C). Two stages were followed in the study. The first stage was conducted experimentally to investigate the tensile and flexural strength of CFRP-strengthened steel plates exposed to service temperatures. The second stage was to develop efficient empirical models using RSM in predicting the tensile and flexural strength of CFRP-strengthened steel plates exposed to service temperatures. The developed model is expected to ease engineers’ calculations of the tensile and flexural strength of CFRP-strengthened steel plates, especially during the preliminary design stage.

## 2. Experimental Program

### 2.1. Methods

In this paper, tensile bonding and flexural bonding were conducted to determine the bond behavior of carbon fiber-reinforced polymer (CFRP)-strengthened steel plates using epoxy adhesive exposed to service temperatures [20,21]. Response surface methodology (RSM) software was used to design the experiments [22,23]. A total of 17 runs of experiments with five repetitive runs were generated by using a Box–Behnken design (BBD). The independent variables were bond length (40–120 mm), the number of CFRP layers (1–5 layers) and temperature (25–70 °C), while the dependent variables were tensile strength and the flexural strength of CFRP-strengthened steel plates. Each experiment was fabricated based on the test specification, and the specimens were named in the form of 40-3-25, wherein the first value stands for bond length, the second value refers to CFRP layers and the last value represents service temperature. After completing the hands-on experiment, the results were re-input into RSM for an optimization analysis to formulate the best-fitted model that is well-suited to the behavior of the result. A field emission scanning electron microscope (FESEM) analysis was conducted after the experiment to determine the microstructure behavior of the composite at different service temperatures.

### 2.2. Material Properties

S275JR mild steel plates (Hong Lam & Co., Perak, Malaysia), unidirectional carbon fiber sheet and epoxy adhesive were used to fabricate the specimens for both experiments. A mild steel plate with a thickness of 5 mm was used. CFRP was prepared through a wet lay-up method. Epoxy resin was prepared by mixing the resin (white) and hardener (grey) in a ratio of 4:1 by weight at its glass transition temperature ($T_{g}$) of 50 °C. The mechanical properties of the CFRP sheet, steel plate and epoxy adhesive were determined in accordance with ASTM D3039 [24], ASTM E8/8M [25] and ASTM D638 [26], respectively. The properties of the materials are tabulated in Table 1.

Prior to fabrication, the steel plates and CFRP surfaces were cleaned with acetone to remove the oil, grease and dust. Surface preparation was crucial because it greatly affects the bonding performance of two different interfaces due to impurities. To ensure bonding quality, the application of epoxy resin and CFRP sheet on the steel plates must be carried out within 2 h of the cleaning process.

### 2.3. Specimens Preparation and Testing Procedures

#### 2.3.1. Tensile Bonding (Double Strap Joint)

**Figure 1:**
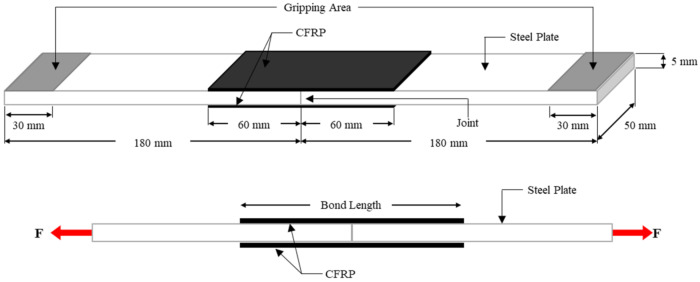
Configuration of double strap joint test.

A double strap joint was used to determine the tensile strength of CFRP-strengthened steel plates. Two steel plates with dimensions of 180 mm × 50 mm × 5 mm were aligned end-to-end after surface preparation. A thin layer of epoxy resin was smeared on the bonded area of the steel surfaces using a brush. Then, a layer of CFRP sheet with a width of 50 mm was overlapped on the epoxy resin. Another layer of epoxy resin was then applied on the CFRP sheet to ensure the CFRP sheet was fully impregnated with epoxy resin. This process was repeated until the desired number of CFRP layers were attached on the steel plates. Lastly, a roller was used to uniformly level the CFRP to remove the air bubbles and excessive epoxy resin and to compact the interfaces. Because of this process, the epoxy adhesive could provide better interlocking between the steel surface and the voids from the CFRP sheet to transfer the loads [27]. The specimen was then left to cure at room temperature for at least 24 h. Then, another side of the steel was applied to CFRP sheet through the aforementioned process. After 14 days of curing, the specimens were tested using a 100 kN capacity universal testing machine (UTM) under static loading. The top and bottom of the specimen were gripped for 30 mm at each end. The specimen was then loaded with a loading rate of 2 mm/min until failure as illustrated in Figure 1.

#### 2.3.2. Flexural Bonding (Four-Point Loading)

**Figure 2:**
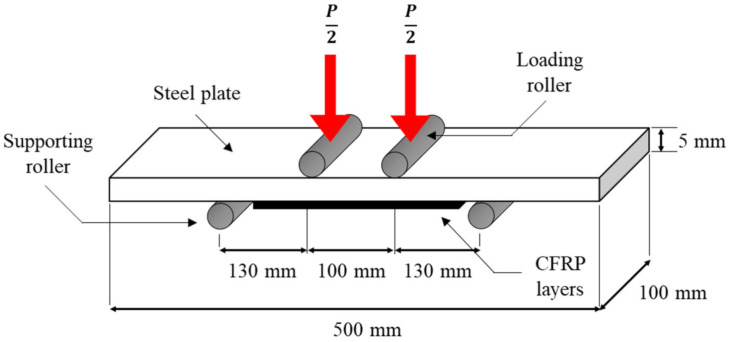
Configuration of four-point load testing.

Flexural bonding was carried out with four-point loading. A steel plate with dimensions of 500 mm × 100 mm × 5 mm was prepared. The procedure for the specimen fabrication was similar to the tensile bonding specimen with 100 mm CFRP width. The steel plate was expected to experience bending in sagging by applying the axial loads to the steel plate; therefore, CFRP layers were installed at the tension area only, as shown in Figure 2. After 14 days of curing, all flexural specimens were tested using a 500 kN capacity UTM. The test was conducted in accordance with the requirements of ASTM 6272 [28] with a static loading at rate of 5 mm/min until failure occurred.

#### 2.3.3. Environmental Exposure

**Figure 3:**
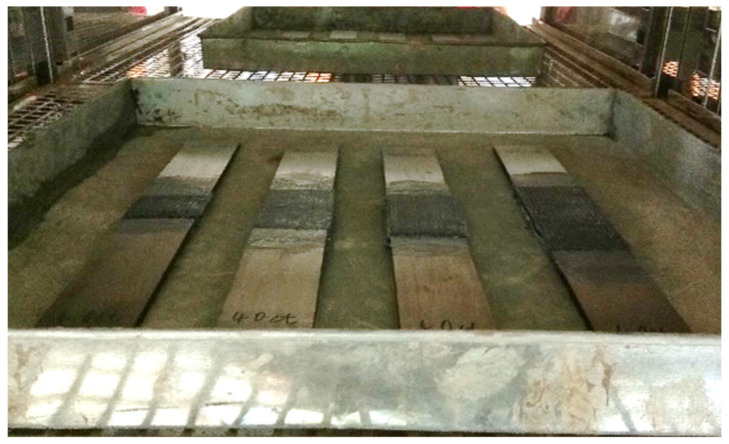
Temperature exposure in environmental chamber.

The aim of environmental exposure was to demonstrate that the specimens were exposed to the service temperature. As the $T_{g}$ of the epoxy adhesive was 50 °C, three different temperatures: room temperature (25 °C), temperature below $T_{g}$ (45 °C) and temperature above $T_{g}$ (70 °C), were selected to investigate the bonding effect of CFRP-strengthened steel plates at different service temperatures. All tensile and flexural specimens were cured for a total 14 days, in accordance with the Sika manual [29]. The first 7 days were spent being cured under room temperature, as recommended by the manufacturer to achieve optimum strength. The remaining 7 days were spent exposed in the oven under the service temperature. After 14 days, the specimens were taken out from the oven and tested immediately. Figure 3 shows the specimens exposed in the environmental chamber.

## 3. Results and Discussion

### 3.1. Failure Modes Due to Tensile and Flexural Bonding

**Figure 4:**
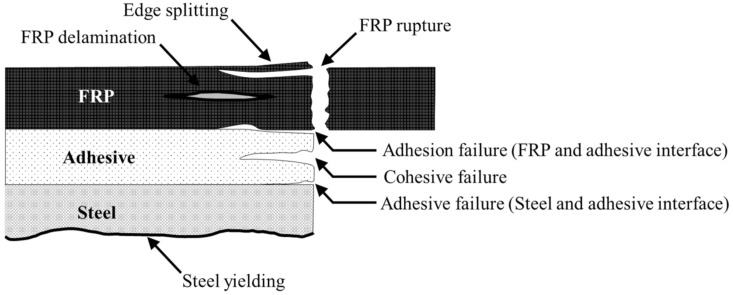
Schematic view of failure modes [31].

Various failure modes may occur in carbon fiber-reinforced polymer (CFRP)-strengthened steel plates, such as adhesion failure at the steel and adhesive interfaces, adhesion failure at the CFRP and adhesive interfaces, cohesive failure, FRP rupture, FRP delamination, CFRP edge splitting and steel yielding, as shown in Figure 4. Adhesion failure is a common result of the chemical and mechanical interactions of the composites. On the other hand, material failure is usually found to be caused by CFRP or adhesive properties [30].

In tensile bonding, the specimens are designed to fail by the separation of two steel plates. When an applied load approaches the ultimate load, a loud cracking sound can be heard, indicating the initiation of debonding and, in a short while, sudden failure occurs through the separation of the adherends. Despite both sides of the steel plates being strengthened with CFRP layers, the failure on both sides does not occur precisely at the same time. Hence, both sides might experience different types of failure modes. However, after one side has failed, the other side will fail shortly, and the first failure is commonly the dominant failure [32]. On the other hand, the failure of flexural bonding is slightly different from tensile bonding as tabulated in Table 2. Tensile bonding specimens are subjected to axial tensile loads that pull apart the specimens, whereas the flexural specimens are subjected to bending loads. The strengthening layers that adhere at the soffit of the steel plate have undergone stretching forces. It is expected that the specimens fail in sagging, which triggers and increases the tendency of the strengthening layers to debond when it reaches its ultimate strength capacity.

**Figure 5:**
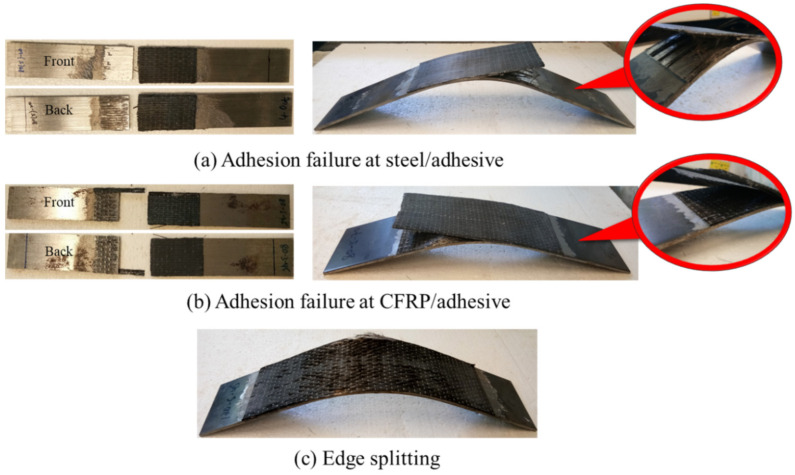
Failure modes.

It is noteworthy that the dominant failure mode of tensile bonding and flexural bonding have been found to be at adhesion interfaces. At room temperature, the debonding tends to occur between steel and adhesive interfaces. The strengthening layers are peeling off from the steel and leave a clear and smooth steel surface, as shown in Figure 5a. However, the failure mode changes when the temperature increases. Service temperatures at 45 and 70 °C shift the adhesion failure to CFRP and adhesive interfaces, as shown in Figure 5b. This is due to the change in the intrinsic mechanical properties of the epoxy resin when service temperature increases. The resin matrix transforms from brittle and glassy to rubbery. The chemical reaction and the formulation of epoxy resin and service temperature cause the resultant effect and the failure mode and therefore, result in the change of failure modes [33,34].

In tensile specimens, some fracture fibers and epoxy residuals were left on the steel plates. This situation indicates that the fibers undergo rupture at higher service temperature when the tensile load is greater than the strengthening layer. In other words, it is also implied that CFRP optimized its full strength before failure. Meanwhile, most of the failures in flexural bonding occurred by CFRP edge splitting at the midspan of the steel, as shown in Figure 5c. At the midspan, the strengthening layers have been stretched outwards and spread along the beam to withstand deflection. The strengthening layers tend to elongate and deform in order to comply with the deflection of the steel, leading to high stress concentration in the strengthening layers and fibers. Owing to the good and strong bonding at both ends of the strengthening layers, the fibers fail by splitting and fracture, especially at the edge of the steel plate, as the load increases. This suggests that the material has fully utilized its strength capacity in strengthening the steel plate. Table 2 also shows that the failure of flexure specimens is mainly found with CFRP edge splitting, regardless of the CFRP layers. Whereas the tensile failure modes of one and three CFRP layers tends to fail in adhesion failure and delamination, for five CFPR layers, it experiences adhesion failure and FRP rupture. It is notable that the strengthening layer does not peel off entirely even when it has reached its ultimate strength capacity. This proves that CFRP-strengthened steel plates are highly reliable, as they do not cause premature or sudden failure when flexural-strengthening steel structures.

### 3.2. Tensile Bonding Results

#### 3.2.1. Ultimate Load Capacity versus Displacement

**Figure 6:**
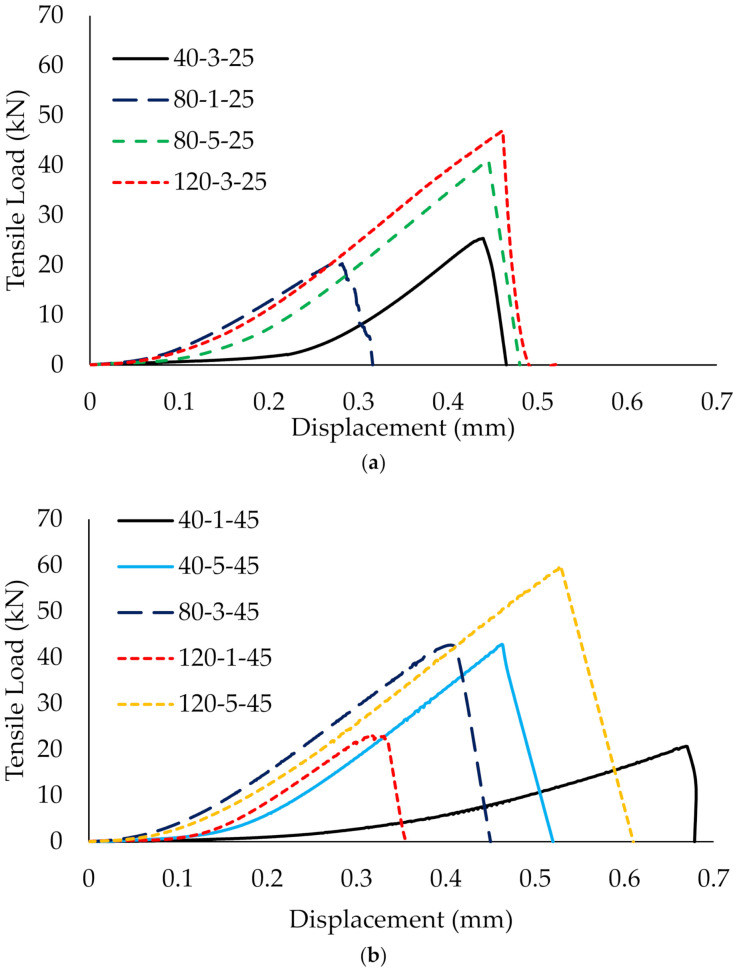

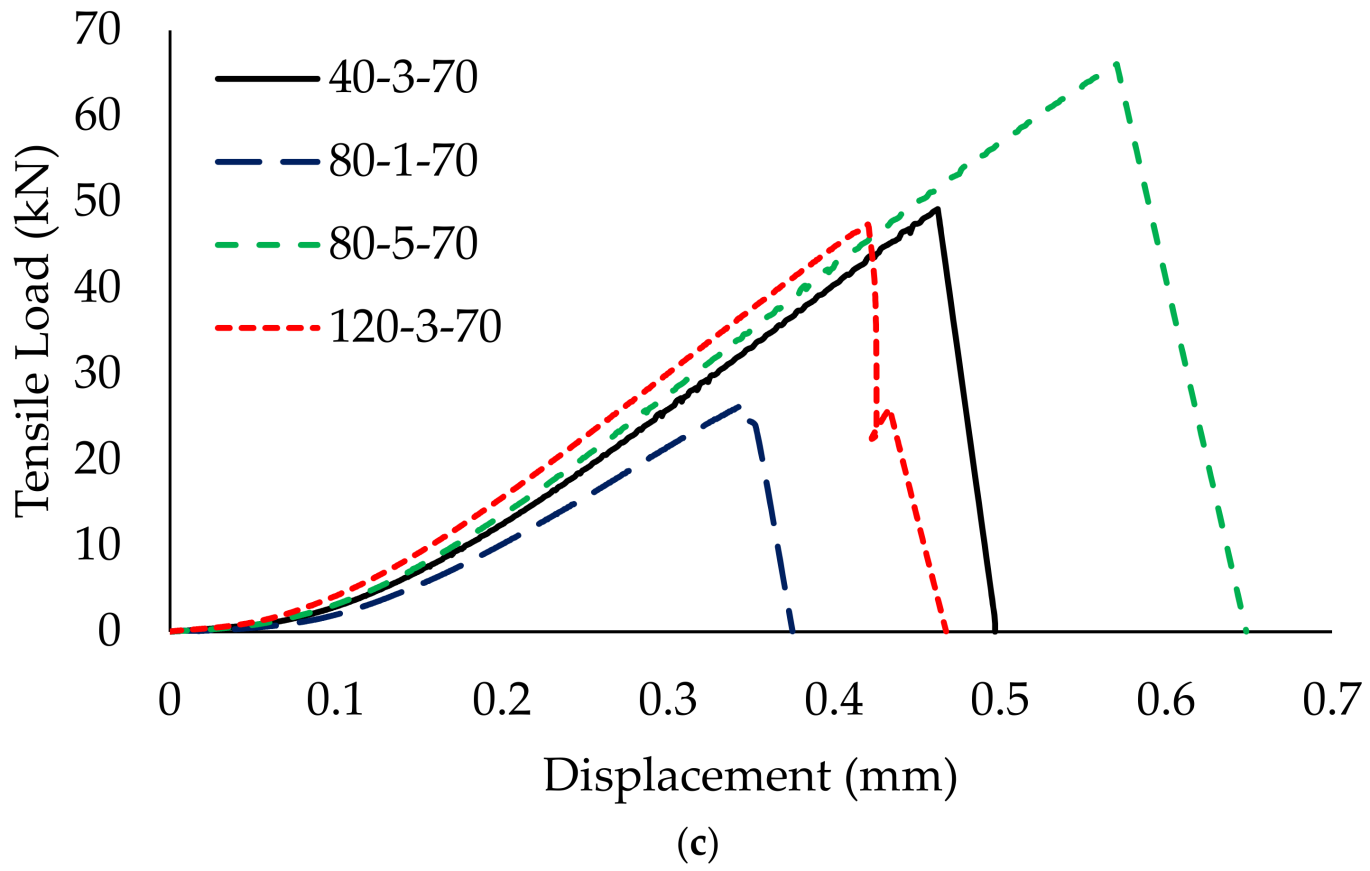
Tensile strength of CFRP-strengthened steel plates at (**a**) 25 °C, (**b**) 45 °C and (**c**) 70 °C.

A total of 17 sets of experiments were tested under double shear lap testing. Figure 6 shows that all graphs behave in a similar manner at 25 °C, 45 °C and 70 °C. The ultimate load increases with displacement at bonded joint. As the load increases, debonding initiates when the load approaches the peak load. Then, a drastic drop of the curve to zero with a loud cracking sound indicates the failure of the specimen. At this point, the debonding of the composite from the steel plates has occurred at the joint of two plates, and the specimen is no longer able to sustain the load any further. This is attributed to the high peeling stress and high shear stress at the brittle fracture of the interfaces [35,36]. Although the change in the failure mode is significant as service temperature increases, the load versus displacement graphs behave in a similar manner even when the service temperature exceeds the $T_{g}$ of the adhesive.

Figure 6a,b shows that the slopes of the curves are inconsistent at 25 °C and 45 °C. This is attributed to the number of CFRP layers and the bond length. As the number of CFRP layers and the bond length increase, the slope of the curve increases. The slope is also connected to the stiffness of the specimen. As the stiffness increases, the energy absorption increases, which subsequently increases the ultimate load capacity of the specimen. However, Figure 6c shows that the slopes of the curves propagate in line with each other when the service temperature reaches 70 °C, regardless of the number of CFRP layers and the bond length. This suggests that, when the service temperature is greater than the $T_{g}$, the adhesive has undergone a softening effect and formed a more homogenous adhesive, so that the composite behaves in a more uniform manner. This also subsequently improved the ultimate load for the specimens, as compared to the specimens at 25 °C and 45 °C.

In addition, the specimens strengthened with three or five layers of CFRP display greater displacement compared to specimens strengthened with one layer of CFRP. The multilayer composites provide higher energy absorption to sustain a higher load before failure, thus, they can withstand larger displacement, while the specimen strengthened with one CFRP layer tends to lose its grip when it starts debonding, due to the brittle adhesive, resulting in failure at low displacement.

#### 3.2.2. Bond Stress

According to Al-Mosawe, et al. [37], the properties of the double strap joint test depends on the adhesive properties because most failures occur within the adhesive layer. Despite this, the ultimate load capacity of all specimens varies even when the failure modes behave the same. Hence, bond stress at the bonded joint can be used to describe the stress of the adhesive at the bonded region. Bond stress, τ, is achieved by:

(1) τ=F/A

where F is the tensile load, and A is the bonded area. A can be calculated by multiplying the CFRP joint width and bond length [38].

**Figure 7:**
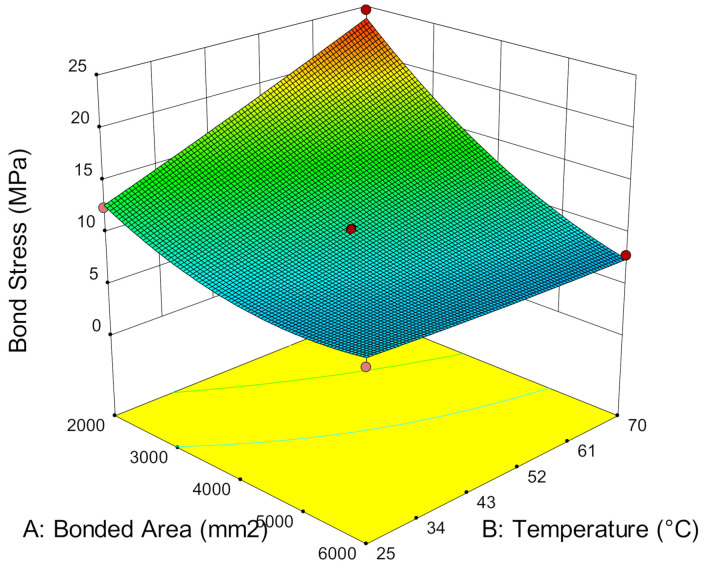
Bond stress versus bonded area and service temperature.

Figure 7 shows the relationship between bond stress and bonded area at different temperatures. It is demonstrated that the lower the bonded area, the higher the bond stress. The concentrated stress which transfers via steel plates to the strengthening layers is distributed along the bonded CFRP. As the width is fixed at 50 mm, the low bonded area is also meant to have shorter bond length and has restricted the stress to transmit due to the limited surface area. Thus, the bonded area has to withstand higher bond stress to sustain the load, whereas in a larger bonded area, the stress can be distributed along the bond length due to the large surface area. Hence, it can reduce bond stress.

Furthermore, Figure 7 also shows that service temperature can influence the bond stress. At low bonded areas, the higher the temperature, the higher the bond stress. The bond stress at 25 °C is reduced by 38.05% and 37.53% as the bonded area increases from 2000 mm^2^ to 4000 mm^2^ and 6000 mm^2^, respectively. At 45 °C, a reduction of bond stress to 34.18% and 56.54% occurs. When the service temperature reaches 70 °C, a high reduction of bond stress to 53.06% and 67.88% occurs when bonded with 4000 mm^2^ and 6000 mm^2^, respectively. This shows that low bonded areas experience higher bond stress and that such stress is negligible in large bonded areas due to the high efficiency of stress distribution over larger surface areas. Although existing studies claimed that adhesive strength declines at elevated temperatures, the inelasticity of the adhesive enables the redistribution of the stress away from the joint along the bonded area to sustain higher stress [20]. Therefore, it can sustain much higher load, longer displacement and contributes to higher bond stress at higher service temperatures.

#### 3.2.3. The Effect of Service Temperature, CFRP Layers and Bond Length

**Figure 8:**
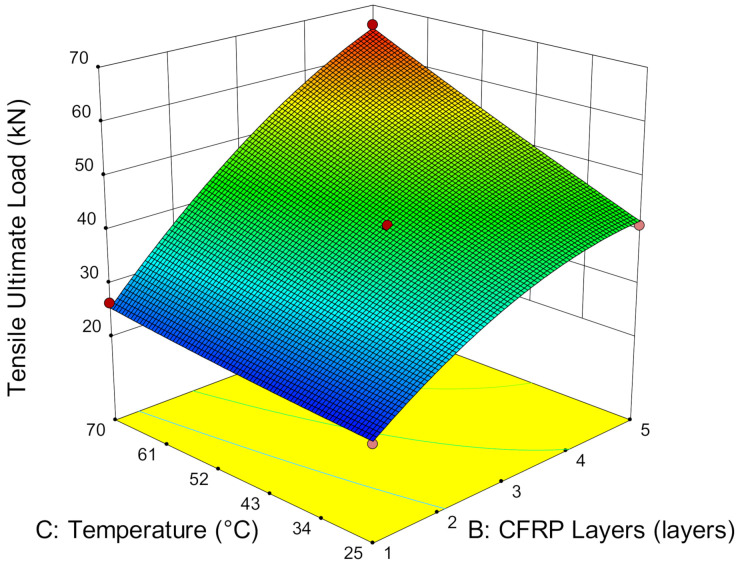
Ultimate load versus temperature and CFRP layers.

Service temperature, the number of CFRP layers and bond length highly affect the ultimate strength capacity of the CFRP-strengthened steel plates. Figure 8 shows that the ultimate load increases with service temperature even when it exceeds the adhesive $T_{g}$. The ultimate load strengthening that a single CFRP layer has achieved at 20.92 kN and 26.28 kN and 25 °C and 70 °C, respectively, is 25.62% strength improvement. Meanwhile, strengthening with five layers of CFRP remarkedly increased the ultimate load capacity by about three times compared to single-layer CFRP, which demonstrates a total of 61.71% enhancement by increasing the temperature from 25 °C to 70 °C. This finding contradicts the existing studies wherein bonding performance did not degrade at high service temperatures, but they did enhance the ultimate load capacity of CFRP-strengthened steel plates. The main difference in our result may be caused by the testing preparation of the specimens that were used in the prior studies and present study [8]. The present study was exposed under service temperatures for seven continuous days prior to testing to demonstrate environmental exposure on the CFRP-strengthened steel structures. Meanwhile, the existing studies that show a reduction in strength were exposed to short-term service temperatures, ranging from just 15 min to 120 min [15,39]. The increase in ultimate strength at service temperature has revealed that the transformation of the adhesive matrix at service temperature does not reduce the bond strength but enables the further enhancement of the interfacial bonding between the steel, CFRP and adhesive interfaces and thus, improves the overall bonding performance.

In addition, it is noteworthy that increases in the number of CFRP layers increases the ultimate load capacity of CFRP-strengthened steel plates. Strengthening with one, three and five CFRP layers successfully improved the strength by, on average, 4.45%, 16.29% and 24.95%, respectively, at 45 °C, compared to room temperature. At a higher service temperature of 70 °C, the strength capacity is further enhanced by 25.62%, 48.79% and 60.71% by strengthening with one, three and five layers, respectively. It has been stated already that a service temperature at 70 °C improved the strength capacity of CFRP-strengthened steel plates more effectively compared to 25 °C and 45 °C. Therefore, the more numerous the CFRP layers, the higher the strength enhancement with respect to the higher exposure temperature.

**Figure 9:**
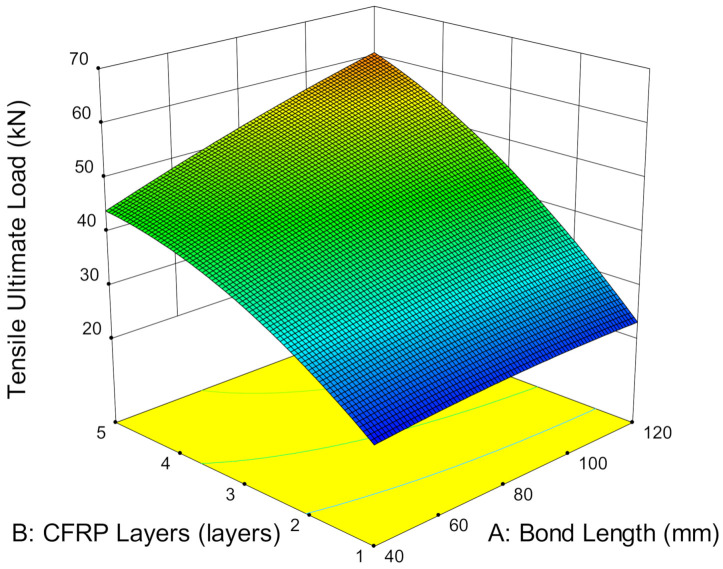
Ultimate load versus bond length and CFRP layers.

Furthermore, bond length, which is relative to the surface area of the strengthening layer, is another crucial factor in determining the strength of CFRP-strengthened steel plates. Figure 9 shows that increasing the bond length from 40 mm to 120 mm using one layer of CFRP can achieve 20.71 kN and 22.99 kN, respectively, which is about 11.01% strength improvement. Moreover, strengthening with five layers of CFRP significantly enhanced the strength capacity by 39.78%, which is more than three times as compared to single layer. The strength attained by 42.83 kN and 59.28 kN uses bond length at 40 mm and 120 mm, respectively. Furthermore, increasing the number of CFRP layers also increases the surface area of the strengthening layers. Figure 9 illustrates that strengthening from one to five layers of CFRP at 40 mm can achieve 106.79% of strength improvement, whereas at 120 mm, the ultimate load capacity increases substantially to 160.38% from one to five layers of CFRP. This suggests that the high stiffness provided by five layers of CFRP enables the strengthening layers to absorb more energy. Besides, the larger surface area of longer bond length also possesses a wider area for stress distribution and reduces the localized stress concentration. Therefore, the combined effects delay debonding failures and improve the ultimate load capacity of the structure.

### 3.3. Flexural Bonding

#### 3.3.1. Ultimate Load Capacity versus Deflection

The flexural bonding of CFRP-strengthened steel plates is another type of testing to determine bonding performance for the adhesive bonding of CFRP. This approach is to generate significant normal stress and have similarly proportioned shear and normal stresses to the larger structures they represent. Although tensile bonding shows that the CFRP composite exhibits a brittle manner, the failure of flexural bonding in ductile behavior is due to the steel. At the beginning, the load increases linearly with deflection in the elastic region. Once the load reaches the yield point, CFRP-strengthened steel plates undergo plastic deformation with sagging deflection. The curves enter the strain-hardening stage accompanied by excessive deflection with an increase in ultimate load [40].

**Figure 10:**
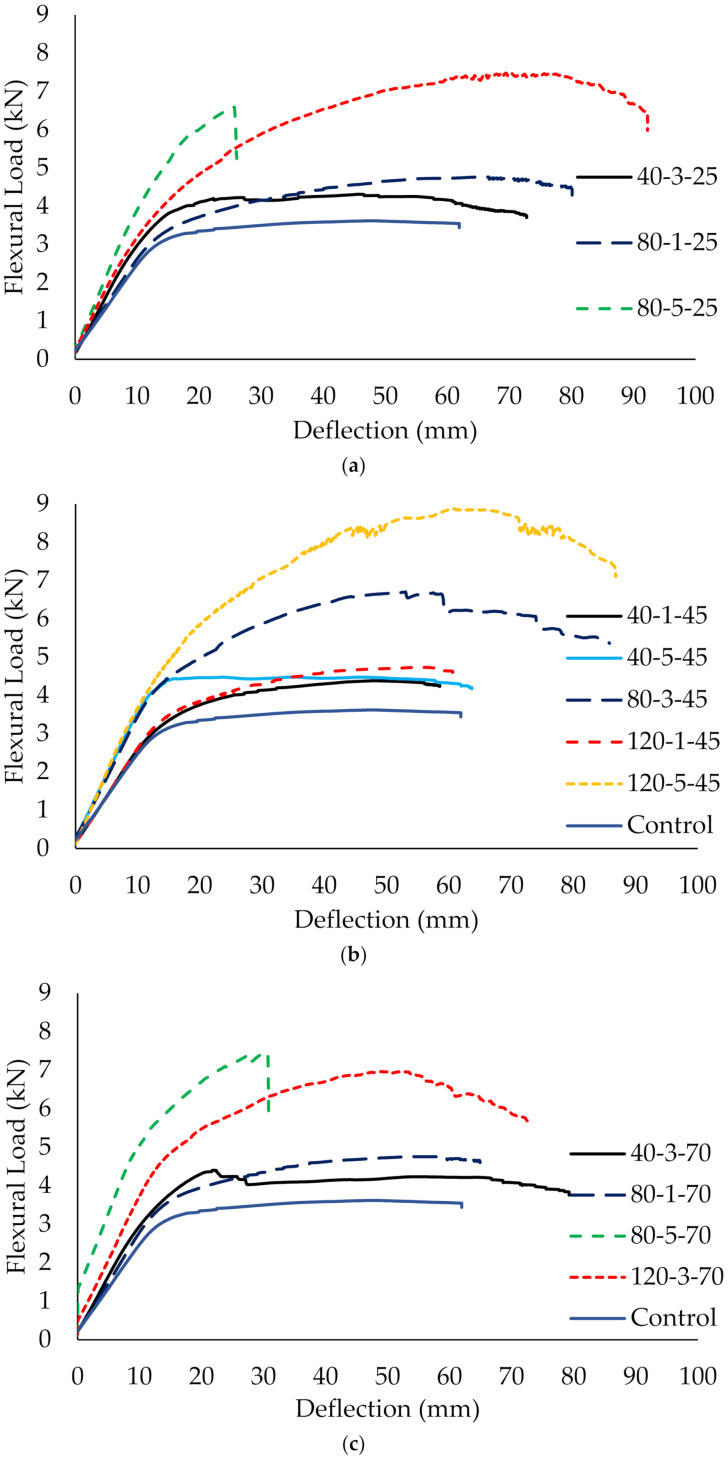
Flexural strength of CFRP-strengthened steel plates at (**a**) 25 °C, (**b**) 45 °C and (**c**) 70 °C.

The load versus deflection graphs show all specimens demonstrate a similar trend regardless of the temperature. For instance, Figure 10 shows that the curves of specimen (40-3-25 and 40-3-70) and (80-1-25 and 80-1-70), with the fixed CFRP layers and bond length, are almost approximated by each other or slightly improve the ultimate strength capacity when exposed to 25 °C and 70 °C. This suggests that the service temperature does not significantly affect the load versus deflection curves. A similar finding corresponds to the results from Sahin and Dawood [20], who tested the CFRP plates-strengthened steel beams at temperature ranging from 25 °C to 50 °C and claimed that the adhesive layer attained adequate stiffness at elevated temperatures in strengthening the composites [20]. This indicates that temperature does not remarkably affect the load versus deflection performance for CFRP-strengthening in flexural members.

However, specimens which were strengthened with five layers of CFRP and an 80 mm bond length (80-5-25 and 80-5-70) showed brittle failure rather than ductile failure. Although a relatively high ultimate load was achieved by the specimens, the load fails abruptly and reduces approximately 30% of the deflection, as compared to unstrengthened steel. The high stiffness provided by five layers of CFRP limits the bending and causes immediate failure to the plate, especially when 61% (80 mm) of the CFRP strengthens between the loading and supporting steel. This load versus deflection behavior is consistent regardless of the temperature. Therefore, it is not recommended to strengthen with five layers and an 80 mm bond length of CFRP due to safety concerns.

In addition, it has also been found that CFRP layers effectively influence the load-bearing capacity of CFRP-strengthened steel plate compared to temperature and bond length. Particularly for a single layer of CFRP, increasing the bond length was shown to be ineffective in improving the curves (40-1-45 and 120-1-45), whereas, as the number of CFRP layers increases, the curve of the load increases substantially even when it has reached the plastic region. Increments in CFRP layers also increase the stiffness of the strengthening layer, forming a higher slope of the curves. However, a single layer of CFRP-strengthening plate behaves similarly to the control (unstrengthened) plate regardless of the bond length. The strength increases marginally and reaches a plateau under a plastic state as the specimen elongates. It is noteworthy that the ultimate load capacity of the strengthened plate is higher than the unstrengthened plate.

#### 3.3.2. Bending Moment, Resilience and Toughness

**Figure 11:**
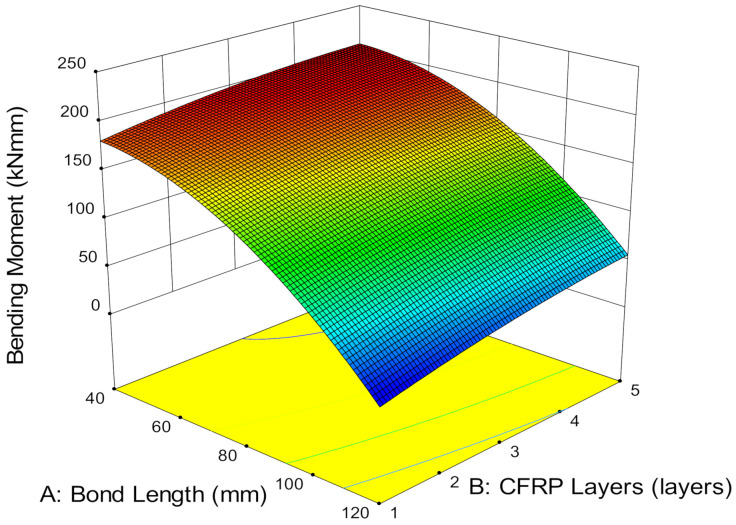
Bending moment versus bond length and CFRP layers.

In flexural specimens, the bending moment is associated with the bond length. The bond length in flexural members is defined as the distance from one of the load points to the end of the CFRP sheet in a region of constant shear force and decreasing bending moment towards the end of the CFRP sheet [41]. Figure 11 observes that increases in bond length reduce the bending moment near the composite end. This is because, when the composite end is far away from the midspan, it provides greater surface area for stress distribution, reduces the tension force caused by the applied load and consequently, increases the ultimate load capacity of the CFRP-strengthened steel plates. Hence, it lowers the bending moment with a longer bond length [42]. However, a high bending moment triggers near the composite end and induces high concentrated stress when the bond length is short. The short distance between the loaded point and CFRP engages higher tension and localized force and thus, leads to failure.

In addition, CFRP layers have been found to increase the bending moment of the specimen. The higher the number of CFRP layers, the higher the bending moment. The high stiffness caused by multiple CFRP layers induces higher energy absorption due to a larger surface area and leads to higher bending moments. Although increasing the CFRP layers at a 40 mm bond length increases relatively low bending moment by 2.26%, it is worth noting that increasing to five CFRP layers at 120 mm bond length achieved a comparatively lower bending moment than a 40 mm bond length. Therefore, longer bond length is favorable in lowering the bending moment of CFRP-strengthened steel plates.

**Figure 12:**
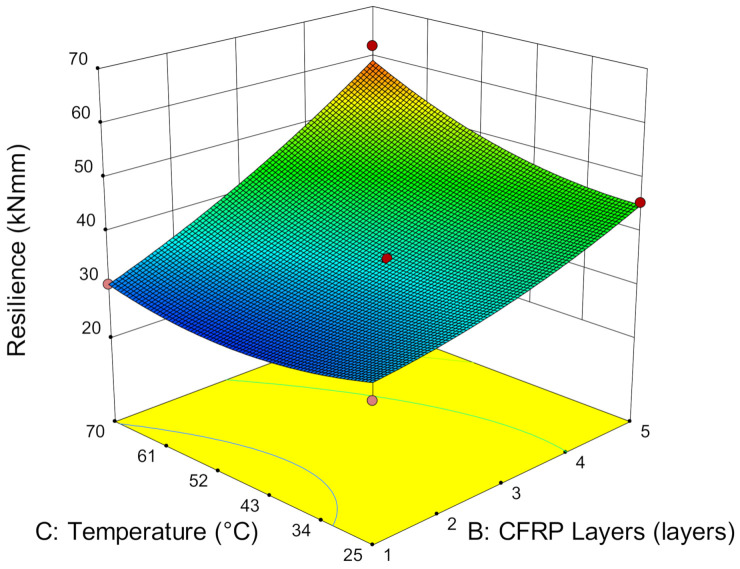
Resilience versus service temperature and CFRP layers.

As service temperature does not significantly affect the load versus deflection curves, it also marginally increases the resilience. Resilience is the energy absorption of deflection in the elastic region and is calculated using the area under the load versus the deflection curve in an elastic state. Figure 12 shows that resilience is quite consistent at every temperature when strengthened with one layer of CFRP. As the number of CFRP layers increases, the resilience increases substantially. A minimum of 58.76% of resilience improvement occurs by strengthening from one to five layers of CFRP from 25 °C to 70 °C. This has again demonstrated that the adhesive layer does not degrade and reduce the adhesive elasticity even when it has exceeded the adhesive temperature.

**Figure 13:**
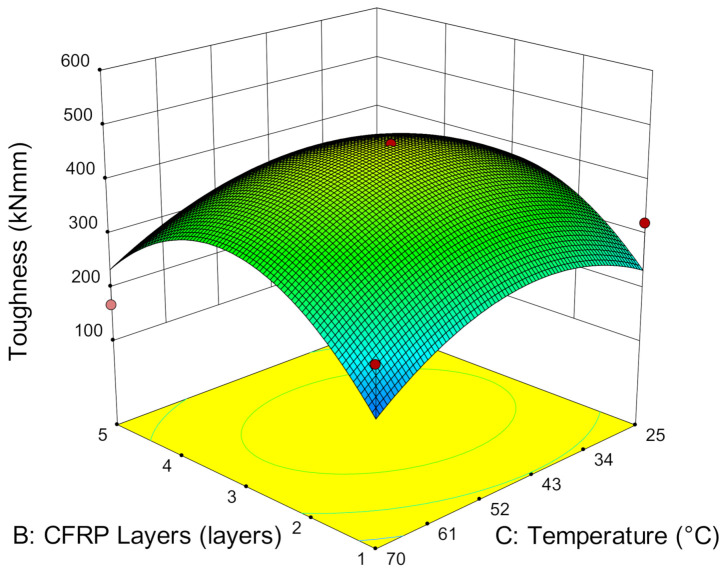
Toughness versus CFRP layers and service temperature.

During the elastic state, the dominant material in sustaining the load is steel, and the secondary supporting material is the strengthening layer. This is also the reason for the flexural specimens achieving almost similar resilience when it strengthened with similar CFRP layers. However, after the steel yields, the stiffness is mainly dependent on the strengthening layers. With this, toughness, which defined as the energy absorption of the deflection until a rupture is formulated, is calculated using the area underneath the load versus deflection graphs, which includes the elastic and plastic regions. Figure 13 shows that toughness increases with the number of CFRP layers. As the number of CFRP layers increases, the high stiffness enables the accumulation of greater energy absorption and subsequently prolongs and increases the bending of the specimen at high load-bearing capacity. The increase of the deflection capacity allows the specimen to absorb more energy and delays the failure, thus, increasing the toughness of the specimen.

Although specimens strengthened with five layers of CFRP can achieve relatively high stiffness, resilience and ultimate load capacity, Figure 13 shows that there is a high tendency for the toughness attained to be slightly lower. This is because the amount of energy absorbed for the high stiffness of CFRP layers is as much as the energy released. The high rigidity of the strengthening layers is restricting the specimen from bending and triggers the brittle failure of the strengthening layers. The specimen does not undergo a strain-hardening process and fails at low deflection rate. Despite the high ultimate load capacity, the low deflection reduces the energy absorption in the plastic region and subsequently reduces the overall energy absorption of the specimens. Hence, this has demonstrated that the brittle failure of the specimen notably affects the energy absorption in the plastic region and thus, lowers the toughness, even though it is reinforced with five layers of CFRP. Hence, strengthening with three layers of CFRP has shown much promise in achieving optimum toughness, compared to five layers of CFRP.

#### 3.3.3. The Effect of Service Temperature, CFRP Layers and Bond Length

**Figure 14:**
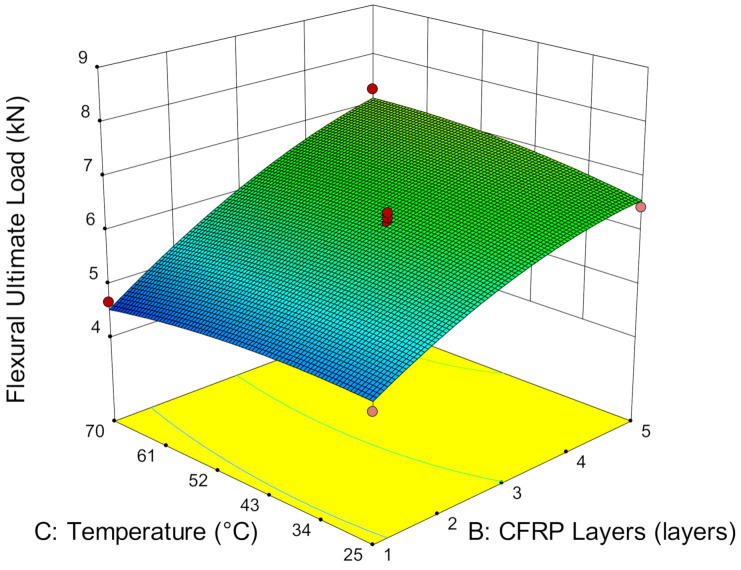
Ultimate load versus service temperature and CFRP layers.

The implications of CFRP flexural bonding steel plate at service temperatures are not obvious. Figure 14 shows that service temperature has minor effects on the ultimate load capacity of CFRP-strengthened steel plates. The ultimate load increases marginally by, on average, only 2.06% as the temperature increases from 25 °C to 70 °C. Particularly when strengthening with one layer of CFRP, the strength difference is relatively small, at 0.17% as the temperature increases. However, the ultimate load capacity increases significantly with the number of CFRP layers. A total of 13.18% strength improvement has been determined when strengthening from one to five layers of CFRP. This is attributed to the multilayers of CFRP and the higher cross-linking of the adhesive molecules as the service temperature increases. Thus, the energy absorption of the composite increases, which subsequently increases the ultimate load capacity of the CFRP-strengthened steel plate. Although service temperature has shown a small effect on the ultimate load in flexural strengthening, it enhances the ultimate load capacity as the CFRP layers increase in number.

In addition, it has been observed that the strengthening layers contributes additional stiffness to the CFRP-strengthened steel plate. An increase in CFRP layers increases the amount of epoxy resin used to bind the composite. The combined effects of the CFRP layers and epoxy resin increase the stiffness of the composite by increasing the energy absorption and subsequently, increases the load-bearing capacity of the CFRP-strengthened steel plate. When the steel plate is strengthened with one layer of CFRP, the ultimate load is attained, on average at 4.57 kN. Strengthening with a single layer of CFRP does not significantly improve the strength. This is because the dominant load of flexural members is mainly sustained by the stiffness of the steel plate. As the number of CFRP layers increase, the ultimate load increases substantially.

**Figure 15:**
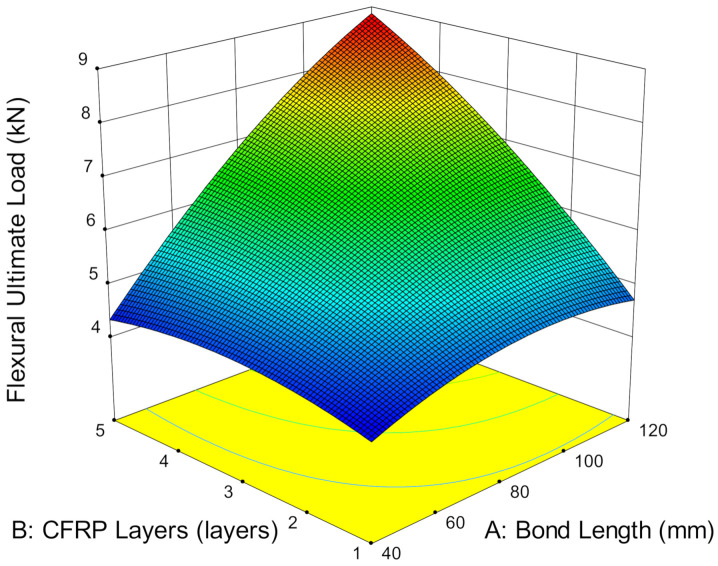
Ultimate load versus CFRP layers and bond length.

Bond length is also associated with the surface area of the CFRP, as well as the bending moment. As the bond length increases, the surface area increases to provide wider area for stress distribution from the loaded point to the composite end and subsequently, reduces the bending moment. The reduction in the bending moment enhances the stiffness of the composite and thus, increases the load-bearing capacity of the CFRP-strengthened steel plate. Figure 15 shows that increasing the bond length from 40 mm to 120 mm increased ultimate load capacity by 7.81% by strengthening with one layer of CFRP. As the CFPR increases to three and five layers, the ultimate load capacity drastically increases as the bond length increases. Both cases show a similar enhancement by increasing on average by 54.15% when the bond length is extended from 40 mm to 80 mm. When the bond length is further enhanced to 120 mm, another 12.19% and 25.40% improvement occurs by strengthening with three and five layers of CFRP, respectively. This is due to the high stiffness of the strengthening layers with respect to its bond length, which increases the energy absorption of the composite and thus, enhances the overall structural capacity.

### 3.4. FESEM Analysis

**Figure 16:**
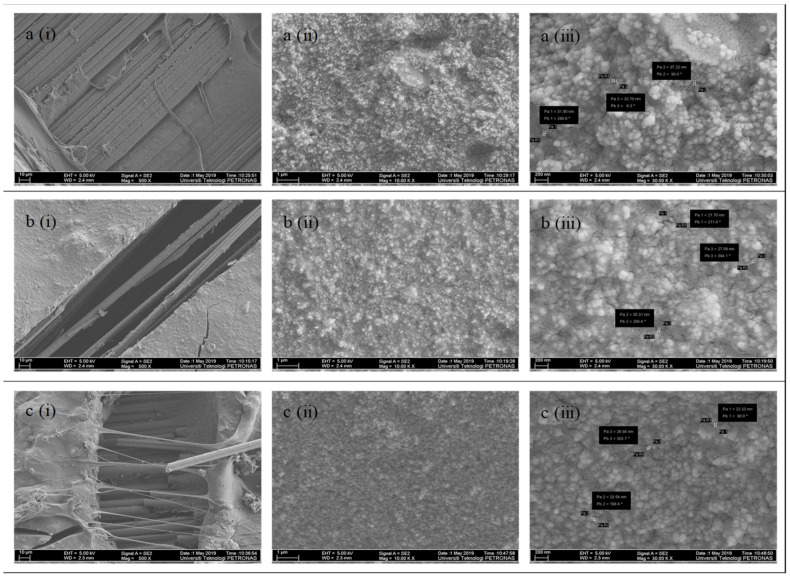
FESEM images at (**a**) 25 °C, (**b**) 45 °C and (**c**) 70 °C under magnification of (**i**) 0.5 kx, (**ii**) 10 kx and (**iii**) 30 kx.

The tested tensile specimens were further analyzed with a field emission scanning electron microscope (FESEM). The microscopy images as shown in Figure 16 were taken at different magnification, such as 0.5, 10 and 30 kx, to investigate the fiber and epoxy resin interfaces exposed to service temperatures of 25 °C, 45 °C and 70 °C, respectively. Figure 16i is focuses on the fiber interfaces under magnification of 0.5 kx, and Figure 16ii,iii reveals the changes in epoxy resin under magnification of 10 and 30 kx, respectively.

At 25 °C, it is observed that the fiber, as shown in Figure 16a(i), remained intact with the epoxy resin. The fibers are aligned in a longitudinal manner like the original CFRP sheet, and the greyish epoxy resin surrounds each individual fibers. This shows that failure occurs through the complete separation of strengthening layers and does not show that much damage occurs on the interface. As the temperature increases, cracks are observed on the epoxy resin, as illustrated in Figure 16b(i). There are also long resin tags at the adhesive layer when the service temperature reaches 70 °C. The long resin has demonstrated the softening effect of the adhesive when it is subjected to service temperature. Furthermore, the fibers have become rounder and more cylindrical than the original flat fibers. This is attributable to the stretching effect of the CFRP sheet and results in the elongation of the fiber. This situation has proven that stress is well distributed along the strengthening layer. The softening effect due to service temperature also contributed to the change in failure mode by transforming from adhesion failure at steel and adhesive interfaces to adhesion failure at CFRP and adhesive interfaces.

Figure 16ii–iii which under magnification of 10 and 30 kx show the detailed formation of epoxy resin under different magnification level. The epoxy resin particles are found to be in a granular shape in a continuous phase at room temperature. The particles, which are made up of bubble-like microstructures, are measured to have an on-average 34 nm gap between the particles. Some interfacial cracks observed surrounding the microstructures are believed to be due to the brittleness of the resin [43]. The interfacial cracks restricted the effectiveness of the stress transfer of CFRP to the bonded joint and caused failure between the steel and strengthening layer. On the other hand, increases in service temperature pushed the microstructures to become denser and more homogenous. The particles have transformed into cotton-like structures and reduced the gaps by, on average, 28 nm and 24 nm at 45 °C and 70 °C, respectively. This has validated the idea that exposure to service temperatures softened the adhesive and minimized the gaps to provide a more compact and stronger composite. Instead of the degradation of the adhesive, service temperatures improved the resin matrix and further enhanced the bonding of the particles by minimizing the particles gap and thus, strengthening the bonding capability of the adhesive.

### 3.5. Response Surface Methodology

Response surface methodology (RSM) is a tool for designing experiments in Design Expert software. It provides functions for mathematical modeling and statistical and optimization analysis. It is also used to design experiments, based on input variables, to reduce the number of experiments. In RSM, the relationship of independent and dependent factors can be established. The input variable is known as the independent factor, and the output variable is known as the dependent factor. The input variables are predetermined based on the collected data from literature reviews and randomly manipulated to design a complete set of experiments. The output variables are the results obtained from the hands-on experiment.

A Box–Behnken interface is a nearly rotatable second-order design based on three-level incomplete factorial designs. It only encounters three levels of each independent factor, including low (−1), medium (0) and high (+1) levels. Although it does not cover all the extreme combinations of all factors, it can provide a precision response at one center point. The tensile and flexural strength of CFRP-strengthened steel plates are computed in Table 3. The design of experiments was generated by the RSM and the results of the tensile and flexural strength obtained experimentally are re-input into the Design Expert software for further analysis.

#### 3.5.1. ANOVA Analysis

In RSM, an analysis of variance (ANOVA) is a statistical analysis to determine the statistical significance of each variable to the proposed mathematical models and analyze the significance of the parameters in affecting the strength of CFRP-strengthened steel plates. All the responses are based on a significance level of 5% (*p* < 0.05) and tabulated in Table 4. The model F-values of 121.44 and 49.89 are considerably high and imply that the models are significant. It means that the responses in the models can be formulated through the regression models for estimating the tensile and flexural strength of CFRP-strengthened steel plates [44]. Moreover, the *p*-values of both models for tensile and flexural strength are shown to be less than 0.05 at the interaction at a 95% confidence level. This demonstrates that the interaction of the factors is statistically significant [45]. Moreover, the lack of fit F-values of 0.62 and 3.69, which are greater than the *p*-value, indicates that the lack of fit is not significant relative to the pure error. There is a 64.02% and 12% chance, respectively, that the lack of fit F-value this large could occur due to noise.

A summary of the model statistics is presented in Table 5. The coefficient of determination (R^2^) is the ratio of the explained variation to the total variation, which shows the goodness of fit for the model [46]. A high R^2^ value, which yields at least 0.8, is considered a good model. Table 5 shows that the R^2^, adjusted R^2^ and predicted R^2^ of tensile strength are 0.9936, 0.9855 and 0.9604, respectively, whereas for flexural strength, they are 0.9846, 0.9649 and 0.8102, respectively. The predicted R^2^ also well corresponds to the adjusted R^2^ with a difference of less than 0.2. All the R^2^ are greater than 0.8, which suggests that the proposed model is in agreement with the experimental result. This illustrates that the models are effective in formulating the tensile and flexural strength of CFRP-strengthened steel plates. Besides, the coefficient of variation (CV) expresses the variation of the experimental response with respect to the predicted responses. The low CV at 3.93% and 4.32% imply the high reliability of the responses. This can be explained because low variation tends to provide fewer differences and more precision results in predicting the responses of the model [44]. This can also be proven through adequate precision, of which the desirability ratio is to be greater than 4 [37]. The adequately high precision values at 36.810 and 25.155, respectively, demonstrate the accuracy of the predicted and experimental responses and thus, can be used to command the design space.

**Figure A1:**
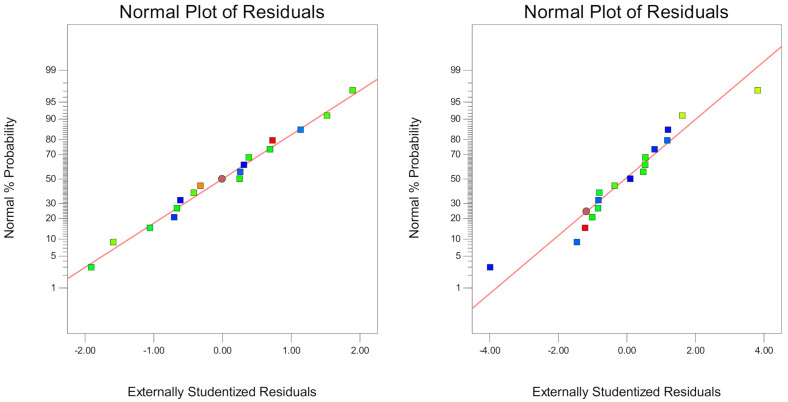
Normal percentage probability plot for the tensile strength (**left**) and flexural strength (**right**) of CFRP strengthened steel plates.

**Figure A2:**
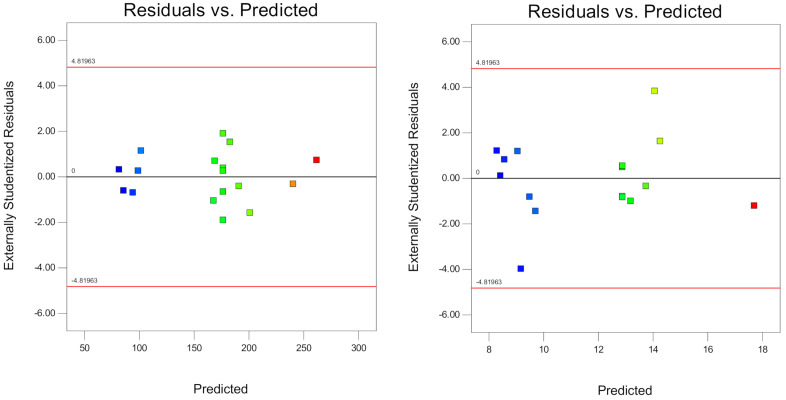
Residual and predicted plot for tensile strength (**left**) and flexural strength (**right**) of CFRP-strengthened steel plates.

**Figure A3:**
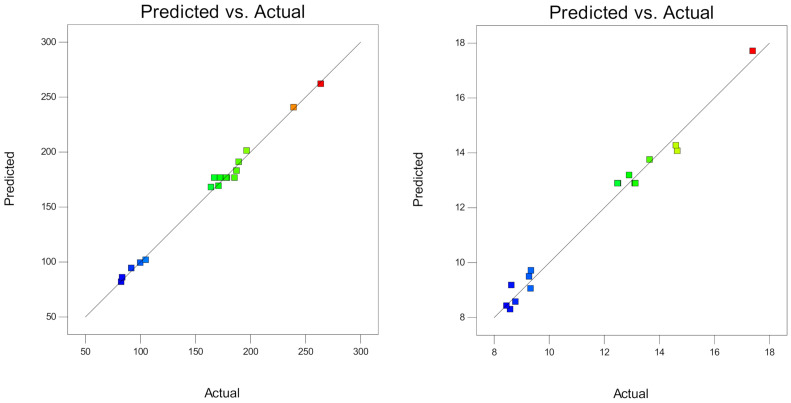
Predicted and actual plot for tensile strength (**left**) and flexural strength (**right**) of CFRP-strengthened steel plates.

To further confirm the reliability of the models, Figure A1, Figure A2 and Figure A3 (as shown in the Appendix A) illustrate the normal percentage probability plot, residual versus predicted plot and predicted versus actual plot of CFRP-strengthened steel plates. In the normal percentage probability plot, the residuals are aligned on a straight line. This shows that the errors are normally distributed and have no serious problem with normality. In Figure A2, all the values are scattered within the plot, indicating no response transformation is required [47]. When the assumptions are satisfied and the modeling is significant, the plot should not exhibit any behavior nor describe any relationship [48]. This is also evidence for the reliability of the model. Additionally, the relationship of the predicted and actual values obtained from the experiment is also discussed in Figure A3. The values are plotted approximately, and the straight line implies well-corresponding predicted values from the proposed model with the actual values from the experiment. This has again demonstrated the adequacy of the model in estimating the tensile and flexural strength of CFRP-strengthened steel plates.

Ultimately, based on the above analysis, the software proposed quadratic types of mathematical models in regression model analyses which well described the relationship between independent and dependent variables in predicting the tensile and flexural strength of CFRP-strengthened steel exposed to service temperatures. The empirical equations are:

(2) $$y_{1}=1.945x_{1}+28.146x_{2}+2.596x_{3}+0.184x_{1}x_{2}-0.026x_{1}x_{3}+0.0434x_{2}x_{3}\;-0.005x_{1}{}^{2}-5.539x_{2}{}^{2}-0.006x_{3}{}^{2}-94.838$$

(3) $$y_{2}=0.107x_{1}-0.106x_{2}+0.096x_{3}+0.025x_{1}x_{2}-0.0003x_{1}x_{3}+0.010x_{2}x_{3}\;-0.0007x_{1}{}^{2}-0.208x_{2}{}^{2}-0.001x_{3}{}^{2}+2.210$$

where *y*_1_ is tensile strength, *y*_2_ is flexural strength, *x*_1_ is bond length, *x*_2_ is the number of CFRP layers, and *x*_3_ is temperature.

#### 3.5.2. Optimization

Multi-objective simultaneous optimization took place in Design Expert software after determining the reliability and adequacy of the regression models. Optimization provides the function to aim for the best solution in acquiring the desired responses. It can evaluate any boundaries of the ranges of variables to achieve either the lowest, medium, highest or any target response [49,50]. The optimized case is assessed through desirability values ranging from 0 to 1. The desirability function is a dimensionless value [39]. Values close to 1 indicate the responses optimized by the software are highly relevant to the experimental study, whereas values close to 0 indicate less relevancy of the optimized responses compared to the experimental study.

**Figure 17:**
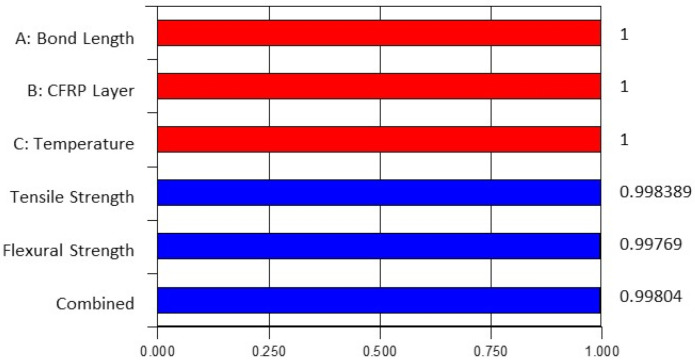
Bar graph of desirability.

**Figure 18:**
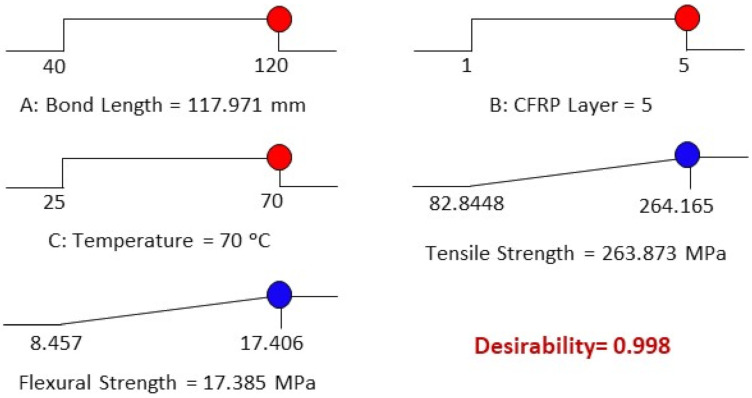
Desirability ramps.

In this study, multi-objective simultaneous optimization is conducted to estimate the bond strength of CFRP-strengthened steel plates. The optimum responses are the maximum tensile strength and flexural strength achieved by varying the independent variables such as bond length, number of CFRP layers and temperature, ranging from 40–120 mm, 1–5 layers and 25 °C–70 °C, respectively. All variables are set within the range values from low to high to achieve the best design. Figure 17 presents the desirability bar graph of independent and dependent variables and their combined desirability, and Figure 18 suggests the optimized case to achieve the optimum strength of CFRP-strengthened steel plates. It suggests that the best design for *x*_1_, *x*_2_ and *x*_3_ are 117 mm, 5 layers and 70 °C, respectively, and the maximum *y*_1_ and *y*_2_ can be attained at 263.873 MPa and 17.385 MPa, respectively. All the desirability values are greater than 0.998, implying the high relevancy of the optimized design to the experimental work.

To validate the optimized design and the proposed model, experimental work was conducted again by repeating the experimental process to determine the tensile and flexural strength of the CFRP-strengthened steel plates. The experiment was conducted by using the variables which were optimized by the software, such as bond length (117 mm), number of CFRP layers (5 layers) and temperature (70 °C) to achieve the approximate result estimated by the models. Specifically, three samples were prepared for each set of specimens, and the average results were recorded after 14 days of curing, which included seven days under room temperature and another seven days under exposed temperature. The outcome shows that the experimental tensile and flexural strength are 275.095 MPa and 17.194 MPa, respectively. The percentage errors of the optimized and experimental tensile and flexural strength are formulated to be 4.16% and 1.11%, respectively. Both errors, which are less than 5%, have validated the correspondence of the experiment values with the predicted values. Thus, it proves the high reliability of the proposed models in estimating the tensile and flexural strength of CFRP-strengthened steel plates.

## 4. Conclusions

In this study, the bond behavior of carbon fiber-reinforced polymer (CFRP)-strengthened steel plates exposed to service temperature were investigated. The results for tensile bonding and flexural bonding were analyzed and optimized using response surface methodology (RSM). The conclusions are as follows:

The failure mode of tensile and flexural bonding occurs at adhesion interfaces. The dominant failure mode at room temperature occurs between steel and adhesive interfaces, and the dominant failure mode at service temperature occurs between CFRP and adhesive interfaces.

1- In tensile bonding, increases in temperature from 25 °C to 70 °C increases the ultimate load capacity by 25.62%, 48.79% and 60.71% after strengthening with one, three and five layers of CFRP, respectively. Increases in bond length also increase strength by about 11.01%. Increasing the CFRP to five layers with respect to the bond length can increase the strength capacity by about three times.
2- In flexural bonding, service temperatures show a mild effect on CFRP-strengthening steel plates compared to CFRP layers and bond length. The ultimate load increases marginally by, on average, only 2.06% as the temperature increases from 25 °C to 70 °C. The ultimate load capacity increases by 2.26% with a 40 mm bond length as the number of CFRP layers increase from one to five, whereas as the CFRP layers increase, using an 80 mm and 120 mm bond length, the ultimate load capacity can remarkedly improve, by 47.39% and 87.61%, respectively.
3- The softening effects of the adhesive at service temperatures reduces particle microstructure gaps to form a densified and homogenous interface and further improve the bonding strength of the adhesive.
4- Two empirical equations with quadratic models are proposed by RSM using a Box–Behnken design (BBD) to estimate the tensile strength and flexural strength of CFRP-strengthened steel plates by considering service temperature, number of CFRP layers and bond length. The optimum tensile strength and flexural strength were achieved by bond length, number of CFRP layers and temperature at 117 mm, 5 layers and 70 °C, respectively.

## Figures and Tables

**Table 1:** Material Properties.

Material Properties	Steel	CFRP	Epoxy Resin
Yield Strength (MPa)	345	-	-
Tensile Strength (MPa)	459	4300	30
Elastic Modulus (GPa)	191.43	225	3.8
Elongation (%)	30	1.91	1.5

**Table 2:** Failure modes of CFRP-strengthened steel plates.

Bond Length (mm)	Number of CFRP Layers	Temperature (°C)	Tensile Bonding	Flexural Bonding
40	3	25	a, e	a, c
80	1		a	f
80	5		a, c	f
120	3		a, c	f
40	1	45	b, e	f
40	5		b, d	b
80	3		b, e, d	b, e
120	1		b, e, d	f
120	5		b, d	f
40	3	70	b, d	b
80	1		b, e	f
80	5		b	b, e
120	3		b, e, d	d

a. Adhesion failure at steel/adhesive interfaces, b. Adhesive failure at CFRP/adhesive interfaces, c. cohesive failure, d. FRP rupture, e. FRP delamination and f. CFRP edge splitting.

**Table 3:** RSM response results.

Standard Order	Run	Bond Length (mm)	Number of CFRP Layers	Temperature (°C)	Tensile Strength (MPa)	Flexural Strength (MPa)
11	1	80	1	70	105.12	9.332
17	2	80	3	45	167.28	12.494
4	3	120	5	45	239.45	17.406
2	4	120	1	45	91.96	9.278
5	5	40	3	25	100.10	8.457
12	6	80	5	70	264.17	14.616
9	7	80	1	25	83.68	9.348
15	8	80	3	45	185.80	12.511
1	9	40	1	45	82.84	8.590
7	10	40	3	70	196.71	8.634
6	11	120	3	25	187.59	14.662
10	12	80	5	25	164.37	12.914
14	13	80	3	45	172.63	13.142
16	14	80	3	45	178.12	13.144
13	15	80	3	45	178.91	13.117
3	16	40	5	45	171.31	8.784
8	17	120	3	70	189.58	13.657

**Table 4:** ANOVA results for tensile strength and flexural strength (quadratic model).

Response	Source	Sum of Squares	df	Mean Square	F-Value	*p*-Value Prob > F	Remark
Tensile Strength	Model	44,486.02	9	4942.89	121.44	<0.0001	Significant
	A-Bond Length	3508.55	1	3508.55	86.20	<0.0001	
	B-CFRP Layers	27,096.29	1	27,096.29	665.71	<0.0001	
	C-Temperature	5538.99	1	5538.99	136.08	<0.0001	
	AB	870.84	1	870.84	21.39	0.0024	
	AC	2216.23	1	2216.23	54.45	0.0002	
	BC	1533.68	1	1533.68	37.68	0.0005	
	A^2^	269.14	1	269.14	6.61	0.0369	
	B^2^	2066.90	1	2066.90	50.78	0.0002	
	C^2^	40.36	1	40.36	0.99	0.3525	
	Lack of Fit	90.00	3	30.00	0.62	0.6402	Not Significant
Flexural Strength	Model	115.81	9	12.87	49.89	<0.0001	Significant
	A-Bond Length	53.01	1	53.01	205.53	<0.0001	
	B-CFRP Layers	35.82	1	35.82	138.86	<0.0001	
	C-Temperature	0.34	1	0.34	1.33	0.2873	
	AB	15.74	1	15.74	61.01	0.0001	
	AC	0.29	1	0.29	1.11	0.3262	
	BC	0.76	1	0.76	2.94	0.1301	
	A^2^	4.50	1	4.50	17.44	0.0042	
	B^2^	2.93	1	2.93	11.34	0.0120	
	C^2^	1.08	1	1.08	4.18	0.0803	
	Lack of Fit	1.33	3	0.44	3.69	0.1200	Not Significant

**Table 5:** Model validation for responses.

Item	Tensile Strength	Flexural Strength
Standard Deviation	6.38	0.51
Mean	162.33	11.77
CV. %	3.93	4.32
R^2^	0.9936	0.9846
Adj R^2^	0.9855	0.9649
Pred R^2^	0.9604	0.8102
Adeq Precision	36.810	24.155

## Data Availability

The data presented in this study are available on request from the corresponding author.
